# Presence and antimicrobial resistance profiles of *Escherichia coli*, *Enterococcus*spp. and *Salmonella*sp. in 12 species of Australian shorebirds and terns

**DOI:** 10.1111/zph.12950

**Published:** 2022-04-22

**Authors:** Hannah G. Smith, David C. Bean, Rohan H. Clarke, Richard Loyn, Jo‐Ann Larkins, Chris Hassell, Andrew R. Greenhill

**Affiliations:** ^1^ Institute of Innovation, Science and Sustainability Federation University Churchill Australia; ^2^ School of Biological Sciences Monash University Melbourne Victoria Australia; ^3^ School of Life Sciences, Centre for Freshwater Ecosystems La Trobe University Wodonga Victoria Australia; ^4^ Institute for Land, Water and Society Charles Sturt University Albury New South Wales Australia; ^5^ School of Science, Engineering and Information Technology Federation University Ballarat Victoria Australia; ^6^ Global Flyway Network Broome Western Australia Australia; ^7^ Australasian Wader Studies Group Broome Western Australia Australia

**Keywords:** antibiotic resistance, enteric bacteria, migratory shorebirds, wildlife

## Abstract

Antibiotic resistance is an ongoing threat to both human and animal health. Migratory birds are a potential vector for the spread of novel pathogens and antibiotic resistance genes. To date, there has been no comprehensive study investigating the presence of antibiotic resistance (AMR) in the bacteria of Australian shorebirds or terns. In the current study, 1022 individual birds representing 12 species were sampled across three states of Australia (Victoria, South Australia, and Western Australia) and tested for the presence of phenotypically resistant strains of three bacteria with potential to be zoonotic pathogens; *Escherichia coli*, *Enterococcus*spp., and *Salmonella*sp. In total, 206 *E. coli*, 266 *Enterococcus*spp., and 20 *Salmonella*sp. isolates were recovered, with AMR detected in 42% of *E. coli*, 85% of *Enterococcus*spp., and 10% of *Salmonella*sp. Phenotypic resistance was commonly detected to erythromycin (79% of *Enterococcus*spp.), ciprofloxacin (31% of *Enterococcus*spp.) and streptomycin (21% of *E. coli*). Resident birds were more likely to carry AMR bacteria than migratory birds (*p* ≤ .001). Bacteria isolated from shorebirds and terns are commonly resistant to at least one antibiotic, suggesting that wild bird populations serve as a potential reservoir and vector for AMR bacteria. However, globally emerging phenotypes of multidrug‐resistant bacteria were not detected in Australian shorebirds. This study provides baseline data of the carriage of AMR bacteria in Australian shorebirds and terns.

**Impacts**

- The AMR profiles of zoonotic pathogens *E. coli*, *Enterococcus* spp. and *Salmonella* sp. isolated from Australian shorebirds and terns was assessed over a 3‐year period (2016–2019). From 1022 individual bird samples, 266 *Enterococcus* spp., 206 *E. coli* and 20 *Salmonella* sp. isolates were recovered.
- AMR was detected in 61% of all recovered bacteria, with 42% of *E. coli*, 85% of *Enterococcus* spp., and 10% of *Salmonella* sp. demonstrating some level of AMR. Resistance to clinically important antibiotic classes, such as the quinolones and aminoglycosides, was detected.
- Resident species were more likely to carry AMR *Enterococcus* spp. than migrant species; however, this pattern of occurrence was not noted for AMR *E. coli* and *Salmonella* sp.

## 1 INTRODUCTION

Antimicrobial resistance (AMR) is recognized as a critical threat to human health (Nesme et al., 2014), increasing the length of stay and mortality risks in patients hospitalized with drug‐resistant infections (Heffernan et al., 2018). Through decades of selection pressures exerted by the misuse of antibiotics in clinical, community, and agricultural settings (i.e. prophylactic dosing, inappropriate prescriptions, antibiotic runoff causing environmental contamination), AMR bacteria have become increasingly common (McGettigan et al., 2019; Wright, 2010). Some bacteria are more capable of acquiring AMR genes than others and, as such, pose a significant risk in clinical settings (Gao et al., 2018). An estimated 700,000 deaths annually are reported worldwide due to antibiotic resistance, and this is expected to rise to 10 million extra deaths annually by 2050 (O'Neill, 2016).

Wild birds are known vectors of zoonotic enteric pathogens that can infect humans (Giacopello et al., 2016; Palmgren et al., 1997; Radhouani et al., 2012), and are reservoirs of enteric bacteria such as *E. coli*, *Enterococcus* spp., and *Salmonella* sp. (Blyton et al., 2015; Santos et al., 2013; Smith et al., 2020). *Enterococcus* spp. have drawn considerable attention due to the impact species such as *E. faecalis* and *E. faecium* have had in clinical settings, being one of the most commonly isolated Gram‐positive nosocomial pathogens in health settings globally (Gao et al., 2018). In addition, *Salmonella* is one of the most common causes of human morbidity and mortality associated with foodborne disease (Chlebicz & Slizewska, 2018).

Migratory shorebirds are potentially an important vector of emerging diseases and AMR due to their highly mobile behaviour. Many Australian shorebird species routinely migrate between the northern and southern hemisphere each year, stopping over on land masses that support approximately one‐third of the global human population on their migrations (Yong et al., 2018). Millions of migrant birds make regular movements between the Artic and Australia (Oldland et al., 2009), and come into close contact with human activity as they do so. Encroaching human development at major stopover sites such as the Yellow Sea and hunting of shorebirds for food increase the amount of contact these migrating birds have with humans (Piersma et al., 2017; Studds et al., 2017; Wauchope et al., 2017). This then increases the likelihood of the transfer of zoonotic diseases and AMR bacteria (Altizer et al., 2011) between birds, humans, and other wild animals.

Information regarding the presence of bacterial species of clinical importance and associated AMR in Australian shorebirds and other highly mobile coastal species such as terns is scarce. Globally, studies concerning shorebirds and terns are lacking, with most investigating the presence of pathogenic Enterobacteriaceae (Keeler & Huffman, 2009; Santos et al., 2012). Hence, it is important to develop baseline measures of AMR in these communities, covering both migrant and resident species. Due to the high number of shorebirds (37 species) and terns (19 species) present, Australia presents an exemplary study site for this purpose (Weller & Lee, 2017).

Here we investigate the presence and AMR profiles of *E. coli*, *Enterococcus* spp. and *Salmonella* sp. as three bacterial species that occur in shorebirds and terns as gut commensals, but with potential to cause disease in humans. Our aim was to quantify the proportions of AMR bacteria in a bird community that contains both migrant and resident species, and to explore factors that may influence AMR carriage in shorebirds and terns.

## 2 MATERIALS AND METHODS

### 2.1 Animal collection and sampling

Twelve bird species were investigated in this study, including ten shorebird species and two tern species (Table 1). All species were caught in tidal habitats on the Australian coast. The shorebirds included three species that breed within Australia; six that breed in Arctic Siberia or Alaska and migrate to Australia for the austral summer; and one that breeds in New Zealand in the austral spring and migrates to Australia for the non‐breeding season (February to September) (Marchant and Higgins 1993; Higgins and Davies 1996). The two terns have resident breeding populations in Australia as well as broad global distributions. Shorebirds were captured by the Victorian Wader Study Group (VWSG), Australasian Wader Study Group (AWSG), and Friends of Shorebirds South East (FOSSE) from December 2016 to February 2019, as part of ongoing scientific and conservation efforts addressing these focal species throughout the East‐Asian Australasian flyway. Birds were captured with the aid of cannon nets, handheld nets, or captured by hand, as appropriate for species. Terns were often captured in the same operations, and were included in this study as they often share habitats with shorebirds, and belong to the same taxonomic order (*Charadriiformes*).

**TABLE 1:** Proportions of bacteria (*E. coli*, *salmonella*sp., and *Enterococcus*spp.) recovered from the ten shorebird and two tern species sampled in Australia

Species (number examined; breeding range; habitats used in Australia)	*E. coli*	*Salmonella*sp.	*Enterococcus*spp.
Crested Tern *Thalasseus bergii* *(n = 95) Aus; Coastal*	68 (72%)	2 (2%)	68 (72%)
Caspian Tern *Hydroprogne caspia* *(n = 89) Aus; Coastal & Inland*	77 (87%)	0 (0%)	78 (88%)
Pied Oystercatcher *Haematopus longirostris* *(n = 31) Aus; Coastal*	4 (13%)	1 (3%)	18 (58%)
Sooty Oystercatcher *Haematopus fuliginosus* *(n = 12) Aus; Coastal*	5 (42%)	0 (0%)	10 (83%)
Sharp‐tailed Sandpiper *Calidris acuminata* (*n* = 131) *Artic; Coastal & Inland*	22 (16%)	0 (0%)	23 (18%)
Sanderling *Calidris alba* *(n = 10) Artic; Coastal*	1 (10%)	0 (0%)	4 (40%)
Red‐necked Stint *Calidris ruficollis* *(n = 86) Artic; Coastal & Inland*	0 (0%)	0 (0%)	20 (23%)
Curlew Sandpiper *Calidris ferruginea* *(n = 180) Artic; Coastal & Inland*	4 (2%)	0 (0%)	19 (11%)
Ruddy Turnstone *Arenaria interpres* *(n = 37) Artic; Coastal*	1 (3%)	0 (0%)	3 (8%)
Bar‐tailed Godwit *Limosa lapponica* *(n = 264) Artic; Coastal*	14 (5%)	17 (6%)	17 (6%)
Double‐banded Plover *Charadrius bicinctus* *(n = 84) NZ; Coastal & Inland*	8 (10%)	0 (0%)	6 (7%)
Red‐capped Plover *Charadrius ruficapillus* *(n = 3) Aus; Coastal & Inland*	1 (33%)	0 (0%)	0 (0%)
Total *(n = 1022)*	206 (20%)	20 (2%)	266 (26%)

*Note:*The proportion of birds tested that were positive for each bacterial species is provided in parenthesis. The breeding locations and habitat preference (coastal or inland) for each species is provided in italics after bacterial proportions.

Birds were sampled in 22 different geographic locations, across the Australian states of Victoria, South Australia and Western Australia. Cloacal swabs were taken from each bird using Mini Tip Amies with Charcoal specimen swabs (Copan). Transit time from sample collection to culture varied, ranging from 9–20 days due to the often‐remote nature of the fieldwork. During transit, swab samples were stored at ~5°C in a portable refrigeration unit.

All research was conducted under animal ethics permits issued by Federation University Australia (permit no. 16‐002), scientific research permits issued by the Department of Environment, Land, Water and Planning for Victoria (DELWP) (permit no. 10008032), the Department of Environment and Water for South Australia (DEW) (permit no. 35/2016), and the Department of Parks and Wildlife for Western Australia (DPaW) (permit no. 01‐000179‐1).

### 2.2 Bacterial culture and antimicrobial susceptibility testing

Swabs were pre‐enriched in 5 ml brain heart infusion broth (Oxoid) and incubated at 35°C for ~24 hr. Aliquots of 100 μl were subsequently used to inoculate each of mannitol broth (Oxoid), azide dextrose broth (Oxoid) and selenite broth (Becton Dickinson [BD]). All selective enrichment broths were incubated at 35°C for 18–48 hr, and then plated onto MacConkey II agar (Oxoid) or xylose lysine deoxycholate (XLD) agar (BD) as appropriate and incubated at 35°C for 24–48 hr.

Suspect *E. coli, Enterococcus* spp. or *Salmonella* sp. isolates were sub‐cultured to purity on the respective selective plates and preliminary testing included Gram reaction, catalase and oxidase testing. Presumptive *E. coli* isolates were confirmed by the indole test. Presumptive *Salmonella* sp. were confirmed by a PCR assay targeting the *invA* gene (Malorny et al., 2003), and presumptive *Enterococcus* spp. were confirmed by a PCR assay targeting the 16 s rRNA gene (Ryu et al., 2013).

Confirmed isolates were tested for antimicrobial susceptibility using the Kirby‐Bauer disk diffusion method, as outlined by the Clinical & Laboratory Standards Institute M100‐S22 (Clinical and Laboratory Standards Institute, 2012). The antimicrobials (Oxoid) tested were dependent upon the bacterial species in question (Tables 2 and 3). Antimicrobial resistance (AMR) was defined as reduced susceptibility to at least one agent tested, while multi‐drug resistance (MDR) was defined as reduced susceptibility to at least one agent in three or more antimicrobial classes (Magiorakos et al., 2012).

**TABLE 2:** Antibiotic susceptibility of *E. coli*isolates recovered from Australian shorebird and tern species

Bird species (no. birds positive)	AK	AMC	AMP	CTX	CAZ	C	CIP	CN	IPM	NA	S	SXT	TE
Crested Tern (*n* = 68)													
Resistant	3	—	3	—	—	—	—	—	—	1	5	1	5
Intermediate	5	1	2	—	—	—	—	—	—	—	11	—	1
Susceptible	60	67	63	68	68	68	68	68	68	67	52	67	62
Caspian Tern (*n* = 78)													
Resistant	2	1	14	—	—	—	1	—	—	—	6	3	13
Intermediate	1	2	4	—	—	1	—	—	1	—	18	—	1
Susceptible	75	75	60	78	78	77	77	78	77	78	54	75	64
Bar‐tailed Godwit (*n* = 14)													
Resistant	—	1	—	—	—	—	—	—	—	—	—	—	—
Intermediate	—	—	1	—	—	—	—	—	—	—	1	—	—
Susceptible	14	13	13	14	14	14	14	14	14	14	13	14	14
Sharp‐tailed Sandpiper (*n* = 22)													
Resistant	—	—	—	—	—	—	—	—	—	—	—	1	1
Intermediate	—	—	4	—	—	—	—	—	—	—	—	—	—
Susceptible	22	22	18	22	22	22	22	22	22	22	22	21	21
Other^a^ (*n* = 24)													
Resistant	2	1	2	—	2	—	—	—	—	2	2	—	—
Intermediate	—	7	—	—	—	—	1	—	1	—	1	—	—
Susceptible	22	16	22	24	22	24	23	24	23	22	21	24	24
Total (*n* = 206)													
Resistant	7	3	19	—	2	—	1	—	—	3	13	5	19
Intermediate	6	10	11	—	—	1	1	—	2	—	31	—	2
Susceptible	193	193	176	206	204	205	204	206	204	203	162	201	185

*Note:*For species with <10 isolates recovered, the total isolates recovered were as such: Pied oystercatcher (*n* = 4), sooty oystercatcher (*n* = 5), red‐capped plover (*n* = 1), curlew sandpiper (*n* = 4), Ruddy turnstone (*n* = 1), sanderling (*n* = 1), and double‐banded plover (*n* = 8).

Antibiotic abbreviations: AK, Amikacin; AMC, Amoxycillin; AMP, Ampicillin; C, Chloramphenicol; CAZ, Ceftazidime; CIP, Ciprofloxacin; CN, Gentamicin; CTX, Cefotaxime; IPM, Imipenem; NA, Nalidixic Acid; S, Streptomycin; SXT, Sulfamethoxazole‐trimethorpim; TE, Tetracycline.

^a^ Bird species for which less than 10 samples were collected.

**TABLE 3:** Antibiotic susceptibility of *Enterococcus*spp. isolates recovered from Australian shorebird and tern species

Bird species (no. birds positive)	AMP	C	CIP	E	CN	S	TE	VA
Crested Tern (*n* = 49)								
Resistant	1	2	4	7	—	—	15	1
Intermediate	—	1	24	36	1	—	—	12
Susceptible	48	46	21	6	48	49	34	36
Caspian Tern (*n* = 49)								
Resistant	11	2	1	1	—	1	4	—
Intermediate	—	3	10	44	—	—	—	1
Susceptible	38	44	38	4	49	48	45	48
Pied Oystercatcher (*n* = 16)								
Resistant	—	—	—	1	—	—	—	—
Intermediate	—	—	3	11	—	—	—	1
Susceptible	16	16	13	4	16	16	16	15
Sooty oystercatcher (*n* = 10)								
Resistant	—	—	—	—	—	—	—	—
Intermediate	—	—	—	7	—	—	—	—
Susceptible	10	10	10	3	10	10	10	10
Bar‐tailed Godwit (*n* = 16)								
Resistant	—	—	—	—	—	1	—	—
Intermediate	—	—	5	6	—	1	—	—
Susceptible	16	16	11	10	16	14	16	16
Red‐necked Stint (*n* = 20)								
Resistant	5	—	—	1	—	—	1	—
Intermediate	—	2	4	15	—	—	—	—
Susceptible	15	18	16	4	20	20	19	20
Curlew Sandpiper (*n* = 14)								
Resistant	—	—	—	—	—	—	—	—
Intermediate	—	—	7	11	—	—	—	—
Susceptible	14	14	7	3	14	14	14	14
Sharp‐tailed Sandpiper (*n* = 18)								
Resistant	—	—	—	1	—	—	—	—
Intermediate	—	—	4	13	—	—	—	—
Susceptible	18	18	14	4	18	18	18	18
Other^a^ (*n* = 14)								
Resistant	—	—	—	—	—	—	—	—
Intermediate	—	—	3	9	—	—	—	1
Susceptible	14	14	11	5	14	14	14	13
Total (*n* = 206)								
Resistant	17	4	5	11	—	2	20	1
Intermediate	—	6	60	152	1	1	—	15
Susceptible	189	196	141	43	205	203	186	190

*Note:*For species with <10 isolates recovered, the total isolates recovered were as such: Ruddy turnstone (*n* = 3), sanderling (*n* = 4), and double‐banded plover (*n* = 7).

Antibiotic abbreviations: AMP, Ampicillin; C, Chloramphenicol; CN, Gentamicin; E, Erythromycin; S, Streptomycin; TE, Tetracycline; VA, Vancomycin.

^a^ Bird species for which less than 10 samples were collected.

### 2.3 Data analysis

Chi‐squared analysis was used in tandem with observed vs expected counts to investigate potential relationships between variables. For chi‐squared analysis, a *p* value of <.05 was considered significant and was indicative of a relationship between the explanatory variable (the species of bird sampled, the feeding ecology of the birds sampled, the habitat of the birds sampled, or the migratory habits of the birds) and the AMR profile of the bacteria recovered. All statistical analysis was performed using SPSS (IBM SPSS Statistics Version 25).

## 3 RESULTS

### 3.1 Sample collection and bacterial recovery

Between 20/12/16–18/02/19, 1022 swabs were collected from ten species of shorebird and two species of tern. From these swabs, *E. coli* was isolated from 20% (206 of 1022), *Salmonella* sp. isolated from 2% (20 of 1022), and *Enterococcus* spp. from 26% (266 of 1022), with all 12 species positive for one or more of the target bacterial species (Table 1).

The three species of bird from which *E. coli* was most commonly detected were the Caspian Tern (88%), Crested Tern (72%) and Sooty Oystercatcher (42%). The same three bird species had the highest proportions of *Enterococcus* spp. (Caspian Terns 88%, Crested Terns 72%, and Sooty Oystercatcher 83%). By contrast, *Salmonella* sp. was only rarely detected, being detected in only 2% of all birds sampled. Of the *Salmonella* sp. isolates, 85% were identified as *Salmonella enterica* serovar Hvittingfoss (*n* = 17, all from Bar‐tailed Godwit, previously reported in Smith et al., 2020), 10% as *Salmonella enterica* serovar Typhimurium (*n* = 2, one from a Crested Tern and one from a Pied Oystercatcher), and 5% as *Salmonella enterica* serovar Bahrenfeld (*n* = 1, from a Crested Tern). Further analysis of what effect variables such as species, feeding and habitat ecology, or migratory movements may have had on bacterial carriage were not possible due to the potential for confounding variables caused by the differing time periods between initial sampling and bacterial culture.

### 3.2 Antibiotic resistance among bacterial isolates

Due to lab restrictions as a result of the COVID‐19 pandemic, not all enterococcal isolates had susceptibility testing performed: in total, 206 of 266 isolates were tested for phenotypic resistance. Overall, AMR was observed in 88 of 206 (43%) *E. coli*, 175 of 206 (85%) *Enterococcus* spp., and 2 of 20 (10%) *Salmonella* sp. In total, 265 of 436 isolates (61%) demonstrated AMR.

For *E. coli*, AMR bacteria were detected in all focal bird species (Table 2). The species with the highest recovery of AMR bacteria was the Caspian Tern, with 45% (*n* = 35) of all *E. coli* isolated from this species demonstrating resistance to at least one antibiotic. Ciprofloxacin resistance was noted in two species (Caspian Tern and Bar‐tailed Godwit) and ceftazidime resistance was noted in two *E. coli* isolates, one each from a Sooty Oystercatcher and a Curlew Sandpiper.

Antibiotic susceptibility testing was performed on 206 of 266 (77%) enterococcal isolates. AMR *Enterococcus* spp. were recovered from all species sampled (Table 3). Vancomycin‐resistant *Enterococcus* (VRE) was isolated from three species (Crested Tern, Caspian Tern, and Double‐banded Plover). The most commonly observed resistance was to the macrolide class to which >70% of enterococcal isolates were resistant. One‐third (32%) of *Enterococcus* spp. demonstrated resistance to ciprofloxacin, and 20 isolates (10%) demonstrated resistance to tetracycline.

Two *Salmonella* sp. isolates demonstrated intermediate resistance against a single antibiotic (streptomycin). These isolates were recovered from a Bar‐tailed Godwit and a Crested Tern. All other *Salmonella* sp. isolates were susceptible to all 15 tested antibiotics.

The number of multi‐drug‐resistant (MDR) strains of bacteria was also investigated. No *Salmonella* sp. isolates were resistant to more than one class of antibiotic. Of *Enterococcus* spp., 17% (*n* = 35) were resistant to ≥3 classes of antibiotics, 4% (*n* = 8) to ≥4, and 1.5% (*n* = 3) to ≥5. Of the *E. coli* isolates, 7% (*n* = 15) were resistant to three or more classes of antibiotics. One percent (*n* = 2) of *E. coli* isolates were resistant to four or more classes of antibiotics.

### 3.3 Bird ecology and antibiotic resistance

A relationship was observed between species of bird and decreased antibiotic susceptibility (defined as any bacteria that demonstrated resistance to one or more antibiotic) and species of bird (*p* = ≤.001). Further analysis revealed that this relationship was only statistically significant for AMR *Enterococcus* spp. (*p* = ≤.001). Due to the low numbers of samples available for individual species, investigation into the relationship between AMR bacteria and each sampled species was not possible. As such, analysis into the impact different ecological variables may have had on AMR carriage was investigated.

The relationship between feeding ecology and the recovery of AMR bacteria was investigated, with birds assigned to one of two feeding guilds. ‘Probers’ (birds that obtain the majority of their food by using their beaks to probe muddy substrates for benthic invertebrates) included the Curlew Sandpiper, Sanderling, Bar‐tailed Godwit, Pied Oystercatcher, Sooty Oystercatcher, and Sharp‐tailed Sandpiper. ‘Peckers’ (birds that gain the majority of their food by hunting on the surface of the sand or mud by sight) included the Red‐necked Stint, Ruddy Turnstone, Double‐banded Plover, and Red‐capped Plover (Crested and Caspian Terns were excluded from this analysis, given they are piscivores). No statistically significant relationship was found between the birds' feeding habits and the recovery of AMR bacteria (of any species).

Next, the relationship between habitat of the birds sampled and recovery of AMR bacteria was investigated. Birds were assigned to one of two habitat guilds‐ ‘Coastal’, which were birds that lived exclusively on the coastline, and ‘Coastal/Inland’, which were birds that utilize both coastlines and inland habitats. No statistically significant relationship was found between habitat guild and the recovery of AMR bacteria (of any species). Additionally, no statistically significant relationship was found between recovery of AMR bacteria (of any species) and the state in which the bird was sampled (either Western Australia or Victoria, with samples from South Australia excluded due to the small sample size).

The relationship between the movement ecology of each species and the recovery of AMR bacteria was also investigated. Birds were defined as ‘migratory’ if they undertake trans‐equatorial migration on an annual basis between high Arctic breeding grounds and non‐breeding grounds in Australia, and ‘resident’ otherwise. Resident birds were more likely to carry AMR *Enterococcus* spp. than migratory birds. Results indicated resident species had significantly higher rates of AMR bacteria resistance than migrant species relationship (*p* ≤ .001) and closer investigation revealed this specifically applied to AMR *Enterococcus* spp. (*p* = .012). No such relationship was detected for AMR *E. coli* (*p* = .259) or *Salmonella* sp. recovery (*p* = .144).

## 4 DISCUSSION

Antibiotic resistance is present in Australian shorebirds and terns, though resistance to clinically important antibiotics and MDR is currently uncommon in the bacteria targeted in this study in wild bird populations. Resistance to clinically important classes of antibiotics such as glycopeptides and carbapenems was rare: less than 8% (*n* = 16) of all *Enterococcus* spp. isolated were resistant to glycopeptides, and less than 1% (*n* = 2) of *all E. coli* isolates were resistant to carbapenems. Notably, all resistance to carbapenems and >90% of glycopeptide resistance was intermediate, rather than complete resistance. Multidrug resistance was uncommon, with 17% of both *Enterococcus* spp. and *E. coli* isolates resistant to three or more classes of antibiotics. MDR was not detected in any *Salmonella* sp. isolates.

Resident birds were more likely to carry AMR *Enterococcus* spp. than migratory birds, though that trend was not apparent in *E. coli* or *Salmonella* sp. Due to their malleable genomes, *Enterococcus* spp. are able to acquire AMR genes with ease. As such, *Enterococcus* spp. may pick up resistance genes from other gut bacteria or the environment to a higher degree than *E. coli* (Ramos et al., 2020). Furthermore, *Enterococcus* spp. originating from faecal contamination (such as that from human sources) have greater persistence in environmental and aqueous environments than *E. coli* (Jin et al., 2004), which could potentially increase the likelihood of human isolates colonizing wild birds. Populations of both *E. coli* and *Enterococcus* are highly variable in wild bird populations (Fogarty et al., 2003) and further studies need to be conducted to confirm if our findings hold true across multiple bird species through different temporal periods.

One explanation for the lower rates of AMR bacteria in migratory birds compared to resident birds as observed here may be due to migratory culling and migratory separation (Altizer et al., 2011). These processes are theorized to curtail parasite and pathogen dispersal among migratory birds as infections negatively impact dispersal (migratory separation) and survival (migratory culling). Positive infection statuses in birds were associated with reduced movement and lowered survival rates (Risely et al., 2018). Despite enterococci being commensal bacteria in birds, previous studies have shown that some enterococcal species can cause disease in multiple species (Devriese et al., 1990; Devriese et al., 1992; Herdt et al., 2009). This may be the cause of the potential relationship seen here‐ migratory birds carrying *Enterococcus* could be less likely to thrive in the long term, while resident birds that do not undergo the same yearly movements are able to tolerate potential infections to a greater degree.

All three target bacterial species (*E. coli*, *Enterococcus* spp., and *Salmonella* sp.) were present in Australian shorebirds and terns. *Enterococcus* spp. and *E. coli* were present in the majority of species sampled, though *E. coli* was not isolated from the Red‐necked Stint. *Salmonella* sp. was present in only three species (Bar‐Tailed Godwit, Crested Tern, and Pied Oystercatcher). The overall isolation proportions of *E. coli*, *Enterococcus* spp. and *Salmonella* sp. in the tested species was 20%, 26% and 2%, respectively. Previous studies examining the presence of bacterial species via genetic sequencing techniques have shown that extended storage times only have a minor negative impact on bacteria present in swab samples; albeit in considerably different experimental settings (Bai et al., 2012; Lauber et al., 2010). While it remains a possibility in our study that bacterial isolation was affected by transport times, it may not have had a major impact on detection rates. Other studies have also found low carriage rates of *E. coli*, *Enterococcus* spp., and *Salmonella* sp. in migratory birds, with *E. coli* having a carriage rate of 1%–9% and *Salmonella* sp. having a 0%–2% carriage rate (Brittingham et al., 1988; Najdenski et al., 2018), similar to this study. While there are no studies that examine the prevalence of *Enterococcus* spp. in migratory birds, studies investigating wild bird populations detected carriage levels of 74%–84% (Marrow et al., 2009; Splichalova et al., 2015). Despite the extended duration between sample collection and culture, the prevalence of target bacteria in this study was broadly comparable to those observed in other studies. It is also important to note that due to the nature of wildlife sampling, some species were caught (and sampled) less often than others, which will affect the proportions of bacterial species recovered. In order to build a more comprehensive view of bacterial prevalence and AMR carriage in wild birds, studies such as this need to be repeated at different time points to account for potential impacts by outside events (such as poor breeding seasons or inclement weather affecting the availability of certain species).

Notwithstanding the similar proportion of culture positive samples in this study relative to previously published studies in birds, culture‐independent approaches could be used in future studies to improve detection of target bacteria. PCR is arguably the best way to determine the presence of target species, given the bacteria does not need be viable to be detected. Thereafter culture could be used for PCR positive samples to obtain isolates for phenotypic AMR testing; or alternatively gene detection by PCR or sequencing could be used. However, there is still some debate around the suitability of gene detection to predict phenotypic AMR, particularly in Gram negative bacteria (Hendriksen et al., 2019; Van Camp et al., 2020). Such challenges may soon be overcome with improved sequencing technologies and better computational approaches to data analysis (Nguyen et al., 2020; Ren et al., 2021).

Contrary to the findings of other studies on Australian birds, AMR in bacteria isolated from Australian shorebirds and terns was more prevalent than previously reported. Overall, 42% of *E. coli* were resistant to one or more antibiotics, as were 85% of *Enterococcus* spp. Resistance in *Enterococcus* spp. was confined mostly to older, broad‐spectrum antibiotic classes such as the macrolides. Among *E. coli*, resistance was evenly distributed among different classes of antibiotics such as the penicillin and aminoglycosides. Vancomycin resistance in *Enterococcus* spp. was found in ≤10% of all isolates, falling between previously reported rates of VRE in wild Australian birds. A study by Oravcova et al. (2017) detected VRE in less than 1% of the sampled birds, while a previous study by members of this research team (Smith et al., 2019) detected vancomycin resistance in 31% of recovered *Enterococcus* spp.

These findings suggest Australian shorebirds and terns harbour bacteria that are sensitive to clinically important antibiotics. This may be due to their ecology; Australian shorebirds and terns do not have significant interactions with anthropogenic or livestock populations, and may only interact with AMR bacteria through environmental sources such as wetland and coastal substrates and water. It is difficult to state with any certainty whether these species acquire AMR bacteria from the environment, as microbiome studies have demonstrated conflicting results. Shorebirds in particular were considered to have a low intake of environmental bacteria (around 2% transfer between environmental bacteria and bird gut microbiota) by Risely et al. (2017), but Grond (2017) suggested that sampling site is the main driver in variation of shorebird gut microbiota. Further studies investigating the genetics and origins of AMR bacteria in Australian shorebird and tern populations are needed to determine where these species acquire AMR bacteria and to provide a more comprehensive assessment of the scope of this problem. Investigations that determine whether migratory birds are capable of transferring AMR bacteria between their own populations, and between human and livestock populations would also be of value.

## 5 CONCLUSION

This study shows that shorebirds are a potential reservoir of AMR pathogens, and are capable of carrying bacteria that are resistant to clinically important antibiotics. This study also shows that migratory status may affect carriage of AMR bacteria, with resident shorebirds demonstrating higher rates of AMR *Enterococcus* spp. than migrant shorebirds. These populations have the potential to act as both hosts and vectors of AMR enteric bacteria. Further studies are required to track AMR in shorebird populations, and to begin to determine how wild populations are acquiring these bacteria.

## CONFLICT OF INTEREST

The authors declare they have no conflicts of interest.

## Data Availability

All new data is present in manuscript.
